# A246 GASTROINTESTINAL ADVERSE EVENTS OF CANNABINOIDS IN PEDIATRIC POPULATIONS: A SYSTEMATIC REVIEW AND META-ANALYSIS

**DOI:** 10.1093/jcag/gwab049.245

**Published:** 2022-02-21

**Authors:** E Ratcliffe, P Karimi, M Sunil, R Leong, J Ramirez-GarciaLuna, G Zuniga-Villanueva

**Affiliations:** 1 Medical Science, Pediatrics, McMaster University Faculty of Health Sciences, Hamilton, ON, Canada; 2 McMaster University Faculty of Health Sciences, Hamilton, ON, Canada; 3 McGill University Faculty of Medicine and Health Sciences, Montreal, QC, Canada

## Abstract

**Background:**

Use of therapeutic cannabinoids in the pediatric population has increased in recent years within areas of epilepsy, palliative care, and cancer. Given the endocannabinoid system plays a crucial role in gastrointestinal (GI) homeostasis, exogenous cannabinoids have the potential to develop GI related side effects. While some GI adverse events have been reported with the use of therapeutic cannabinoids, the full profile of GI adverse events in the pediatric population is still unknown.

**Aims:**

To understand the impact of therapeutic cannabinoids on the GI system of pediatric patients, we performed a systematic review and meta-analysis to assess the prevalence of various GI-related adverse events arising from cannabinoid usage within pediatric populations.

**Methods:**

Searches were conducted from OVID MEDLINE, EMBASE, CINAHL, Web of Science, and The Cochrane Library for study screening. The included studies were quantitatively assessed for GI adverse events including nausea, diarrhea, increased appetite, decreased appetite, weight gain, weight loss, constipation, abdominal pain, and unspecified events. The prevalence along with their relation towards diagnosis, type of cannabinoid, dosage, duration of treatment, and study type were also assessed.

**Results:**

Among a total of 1201 patients across 25 included studies, an overall prevalence of 33.91% GI adverse events was observed in patients. The statistical analysis of the pediatric population displayed no relation between therapeutic cannabinoid usage and GI adverse events, bearing a large heterogeneity (I^2^ = 91%, P < 0.01). Upon analysis based on study type, a significant difference was observed between prospective and retrospective studies in categories of all GI adverse events, diarrhea, and decreased appetite, with prospective studies displaying a higher prevalence in all three categories. Nausea symptoms showed a significant negative correlation with maximum dosage of cannabinoid used. In addition, diarrhea was found to have a higher prevalence in Dravet syndrome related to an ion channel gene mutation (SCN1A) compared to other epilepsy patients.

**Conclusions:**

While the meta-analysis did not identify any significant associations between therapeutic cannabinoid usage and GI adverse events, there remains the potential for interactions of cannabinoids on GI symptoms depending on dosage and underlying patient factors.

**Funding Agencies:**

Farncombe Family Digestive Health Research Institute

